# Laparoscopic Versus Robotic Adrenalectomy: A Randomized Clinical Trial

**DOI:** 10.1245/s10434-025-18567-0

**Published:** 2025-10-25

**Authors:** Eren Berber, Arturan Ibrahimli, Edip Memisoglu, Ege Akgun, Rafael Perez-Soto

**Affiliations:** Center for Endocrine Surgery, Cleveland Clinic, Cleveland, OH USA

## Abstract

**Background:**

This study aimed to compare perioperative outcomes between laparoscopic and robotic transabdominal lateral adrenalectomies.

**Background:**

Despite growing interest in robotic adrenalectomy (RA), its benefits compared with those for laparoscopic adrenalectomy (LA) need to be identified. Two previously published randomized studies used out-of-date technologies and included only pheochromocytoma patients, respectively.

**Methods:**

A prospective randomized clinical trial was conducted by a single surgeon between May 2024 and February 2025. Patients with adrenal tumors eligible for minimally invasive lateral transabdominal adrenalectomy were randomized to LA or RA. The trial was powered to detect a 30-min difference in operative time. The secondary outcomes were perioperative outcomes, cost, and ergonomics, measured by the NASA Task Load Index (NASA-TLX) and the Rapid Upper Limb Assessment (RULA).

**Results:**

In the study, 27 patients were randomized to each group. The groups were similar in demographics, clinical characteristics, and operative indications. The operative times and secondary outcomes were similar between the groups except that the RA group had a lower median operating surgeon NASA-TLX score (16 vs 48; *P ≤* 0.001) and a lower overall RULA score (14 vs 17; *P* = 0.001) than the LA group. Operation room, hospital, and total costs were similar between the groups. Although all the procedures were completed as planned in the RA group, the LA group had four conversions from the original minimally invasive plan (conversion to open, hand-assisted procedure, and partial adrenalectomy and abortion of the procedure in one patient each).

**Conclusions:**

Perioperative outcomes, including cost, were similar between LA and RA, with better ergonomics and fewer conversions from the original minimally invasive surgical plan in the RA group.

**Supplementary Information:**

The online version contains supplementary material available at 10.1245/s10434-025-18567-0.

During the last two decades, many studies have reported the safety and efficacy of robotic adrenalectomy (RA).^1–5^ An analysis of the 2016 National Inpatient Sample data showed that 33% of the adrenalectomies in the United States were performed robotically.^5^ However, despite the popularization of RA, the literature has scant data regarding how it affects patient outcomes compared with laparoscopic adrenalectomy (LA).

The first randomized study comparing RA and LA in 2004 actually showed inferior results with RA in terms of feasibility, morbidity, and cost.^6^ Nevertheless, this study used a first-generation robotic technology, which lacked many features of currently available systems, such as ease of docking and a fourth robotic arm. The second randomized study in 2020 included only patients with pheochromocytoma and did not include docking time in the operative time analysis.^7^

With the growing interest in RA and more frequent incorporation of RA into training programs, a contemporary comparison of this approach with LA is needed. Our group started a robotic adrenalectomy program in 2008 and has described the technique in previous publications.^8,9^ Due to the large adrenal surgical volume, both the laparoscopic and robotic approaches are used on a regular basis in the program. The current study aimed to compare RA and LA using a prospective randomized design.

## Methods

This prospective single-surgeon randomized trial of patients with adrenal disorders, approved by the Cleveland Clinic Institutional Review Board (IRB #23-422) and registered at clinicaltrials.gov (NCT06407024), compared patients undergoing LA with those undergoing RA via the lateral transabdominal approach. The trial protocol is available in Supplement 1. The study followed the Consolidated Standards of Reporting Trials (CONSORT) reporting guidelines.

The inclusion criteria specified patients with a diagnosis of an adrenal tumor/pathology for whom a minimally invasive adrenalectomy was planned at the Department of Endocrine Surgery at the Cleveland Clinic.

The exclusion criteria ruled out (1) patients requiring an open adrenalectomy based on imaging studies suggesting a malignancy with local tissue invasion, (2) patients with a same-quadrant surgical history expected to be associated with extensive abdominal scarring, (3) patients for whom a partial rather than a complete adrenalectomy was planned because the former could be a shorter procedure, (4) and patients for whom a posterior adrenalectomy was planned (patients who had an extensive surgical history with significant intra-abdominal adhesions and those requiring bilateral adrenalectomy).

The primary outcome was skin-to-skin operative time. The secondary outcomes were conversion to a hand-assisted or open approach, conversion to partial adrenalectomy, number of trocars used, estimated blood loss, postoperative pain scores (according to visual assessment scores of 0 [no pain] to 10 [worst pain]), hospital stay, cost, postoperative morphine-equivalent opioid consumption, postoperative 30-day morbidity, surgical margin clearance for malignant tumors and pheochromocytoma, surgeon mental load, as assessed by the National Aeronautics and Space Administration (NASA) Task Load Index (NASA-TLX) scale, and surgeon ergonomics, as assessed by the Rapid Upper Limb Assessment (RULA) survey

### Sample Size Calculation

A review of the adrenalectomy database for minimally invasive lateral transabdominal adrenalectomies between 2015 and 2022 at the Endocrine Surgery Department of Cleveland Clinic showed a mean operative time of 147.4 ± 37.9 min. A sample size calculation using an alpha of 0.05, a beta of 0.2, 50%/50% proportions, a hypothesized 30-min difference in operative time between robotic and laparoscopic approaches using the historical standard deviation of 37.9 showed the need for 27 patients in each group, giving a total of 54 patients. Because the study did not involve follow-up participation other than the immediate follow-up assessment, a subject drop-off component was not added to the sample size.

### Randomization

**Fig. 1:**
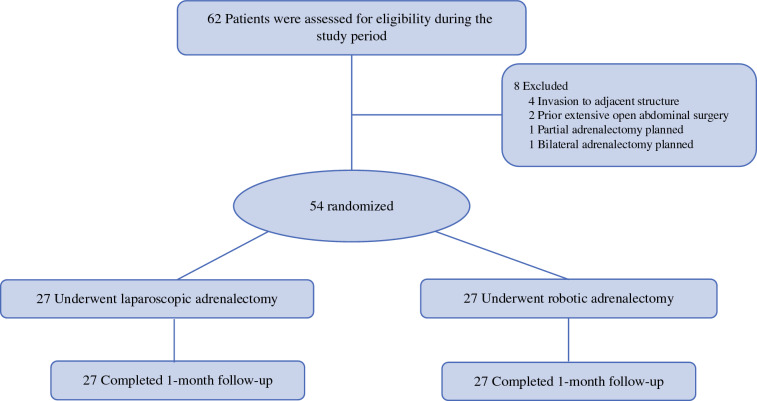
Patient flow diagram of study recruitment.

The patients were randomized by research fellows at least 1 week before the surgery day using Research Electronic Data Capture (REDCap; Vanderbilt University) to allow for optimal scheduling of cases. The initial stratification was performed based on body mass index (BMI), with the patients categorized as either ≥ 35 or < 35 kg/m^2^. Then a block randomization method with a 1:1 allocation ratio was used to assign the patients equally to each treatment group (Fig. 1).

### Study Period

Study recruitment started on 23 May 2024, and ended on 19 February 2025, when the target sample size was reached.

### Surgical Technique

The procedures were performed after general anesthesia with the patients in a lateral decubitus position. The patients received an intravenous injection of a first-generation cephazolin for antibiotic prophylaxis.

#### Right Adrenalectomy

After the abdomen was prepped and draped, a 1.2-cm incision was made about 2 to 3 cm below the right costal margin using an optical trocar. Through a 5-mm periumbilical trocar, a snake retractor was used to retract the liver in both the robotic and laparoscopic cases. In the robotic cases, three 8-mm trocars and a 12-mm trocar were inserted along the costal margin for robotic instruments, including a Cadiere grasper, a Maryland dissector, a 30-degree camera, and a SynchroSeal vessel sealer (Intuitive Surgical Inc, Sunnyvale, CA, USA).

From the right lower quadrant, either a 5- or 12-mm laparoscopic trocar was inserted, depending on the complexity of the procedure, for the first assistant. The right triangular and coronary ligaments were divided to provide exposure to the adrenal gland. In most cases, a vein-last approach was used. The adrenal vein was most frequently divided with the robotic vessel sealer after a 3-0 silk tie  was placed on the caval side.

Indocyanine green (ICG) imaging was performed in each case via a peripheral injection of 5 mg ICG. Laparoscopic or robotic ultrasound was used at the discretion of the surgeon to identify the critical anatomic structures. The specimen was removed outside the 12-mm right upper quadrant port site after placement of a specimen bag.

For laparoscopic right lateral transabdominal adrenalectomy, the trocar placement was similar, with three 5-mm trocars and one 12-mm trocar inserted below the costal margin, as well as a 5-mm periumbilical trocar for the liver retractor. Additional trocars were inserted at the discretion of the surgeon if further retraction or a different angle of dissection was necessary.

The adrenal dissection was performed using a bipolar grasper and Harmonic scalpel. Laparoscopic ultrasound was used at the discretion of the surgeon if the anatomy was not visually evident. The adrenal vein usually was divided after placement of two metallic 5-mm clips on the caval side and one clip on the specimen side.

In both the robotic and laparoscopic cases, a linear vascular stapler was used to manage large adrenal veins (> 7 mm). Specimen extraction was the same as in RA. At the end of the procedure, a laparoscopic transversus abdominis plane block was performed in both RA and LA procedures using 20 ml of bupivacaine liposome suspension (Exparel, Pacira Biosciences, Inc., Parsippany, NJ, USA) with 30 ml of 0.25% bupivacaine and 30 ml of 0.9% NaCl.

#### Left Adrenalectomy

In both groups, the initial entry was performed in the left upper quadrant 2 cm below the costal margin using an optical trocar. In the robotic cases, four trocars (three 8-mm trocars and one 12-mm trocar) were placed along the costal margin for robotic instruments (a Cadiere grasper, a Maryland dissector, a 30-degree camera, and a SynchroSeal vessel sealer). An additional 5- or 12-mm trocar was inserted in the left lower quadrant for the first assistant.

Initially, the splenic flexure of the colon was mobilized, and Gerota’s space was entered in the retroperitoneum. Then, this dissection plane was carried upward to mobilize the spleno-renal ligament and perform a left medial visceral rotation to drop the spleen and pancreas for exposure of the adrenal gland. In each case, ICG imaging was performed via a peripheral injection of 5 g of ICG. Laparoscopic or robotic ultrasound was used at the discretion of the surgeon to identify the critical anatomic structures.

The adrenal vein was managed the same way as in the right side by dividing it with the robotic vessel sealer after placement of a 3-0 silk tie on the staying side. The specimen was removed outside the 12-mm right upper quadrant port site after placement in a specimen bag. For laparoscopic left lateral transabdominal adrenalectomy, the trocar placement was similar to that on the right side, with three 5-mm trocars and one 12-mm trocar inserted below the costal margin. Additional trocars were inserted at the discretion of the surgeon if additional retraction or a different angle of dissection was necessary.

The adrenal dissection was performed using a bipolar grasper and a Harmonic scalpel. Laparoscopic ultrasound was used at the discretion of the surgeon if the anatomy was not visually evident. The adrenal vein usually was divided after placement of two 5-mm metallic clips on the staying side and one clip on the specimen side. Specimen extraction was the same as in RA. At the end of the procedure, a laparoscopic transversus abdominis plane block was performed in both RA and LA procedures.

Both the laparoscopic and robotic adrenalectomies were performed by the attending surgeon (E.B.) and an endocrine surgery fellow. All cases were teaching cases with the fellows participating in the procedures according to their skill levels and case complexity. In the robotic cases, dual-console Xi systems (Intuitive Surgical) were used. In both the laparoscopic and robotic procedures, the attending surgeon took the lead in the procedures, allowing the fellows to perform certain steps of the procedure (i.e., exposure, dissection, hemostasis) in accordance with their skill levels and the complexity of the specific cases.

Postoperatively, an enhanced recovery pathway was followed, with early ambulation and diet, as well as avoidance of narcotics as much as possible. On postoperative day 1, a morning cortisol was obtained in all but the Cushing’s syndrome patients, followed by an ACTH stimulation test for those patients with a serum cortisol level lower than 10 µg/dL. All the patients showing signs of adrenal insufficiency, those with a serum cortisol level lower than 5 µg/dL, or those with a serum cortisol level of 5 to 10 µg/dL but a failed ACTH stimulation test were started on steroids on postoperative day 1.

### Workload Assessment by the NASA Task Load Index Scale

The NASA Task Load Index (NASA-TLX) scale is an assessment tool first introduced by Hart and Staveland in 1988 to evaluate the workload during or immediately after task performance.^10^ It has been increasingly used in surgical ergonomics research, particularly to evaluate minimally invasive surgery.^11–13^

The NASA-TLX captures an overall workload score based on the weighted combination of six distinct subscales, each rated by the respondent on a 0- to 100-point visual analog scale. These six subscales comprise the following:

1. Mental demand: assesses the degree of cognitive activity required, including perceptual and decision-making processes.
2. Physical demand: measures the extent of physical effort and activity needed to complete the task.
3. Temporal demand: refers to the time pressure felt during task completion, evaluating whether the pace was slow and leisurely or rapid and rushed.
4. Performance: reflects self-perceived success or satisfaction in task execution. Unlike other subscales, it is anchored by “good” (low values) and “poor” (high values).
5. Effort: assesses the overall amount of mental and physical energy required to achieve the performance level.
6. Frustration level: Measures emotional response, particularly levels of stress, irritation, or discouragement experienced during the task.

The overall workload score typically is calculated using a weighted-average approach, in which participants first perform pairwise comparisons between the six factors to determine their relative importance, then rate each component individually on the analog scales. In this study. NASA-TLX assessments were performed immediately after the operations by both the primary surgeon and the assistant surgeon.

### Ergonomics Assessment by Rapid Upper Limb Assessment

The RULA survey is a validated ergonomic risk assessment tool developed by McAtamney and Corlett in 1993 to evaluate posture-related musculoskeletal risk, particularly for work-related upper limb disorders.^14^ Originally designed for use in industrial and office environments, RULA has since been adapted to evaluate the ergonomics of surgeons while doing minimally invasive surgery.^15,16^

As an observational tool, RULA evaluates the posture, muscle use, and external forces acting on a subject’s upper limbs, trunk, and legs during task performance. Each part of the body is assessed and given a final score as score A for upper limb and score B for trunk and legs. Then these scores are combined to yield a final score denoted score C or the RULA score (range, 1–7). A final RULA score of 1 to 2 indicates an acceptable posture, with 3 to 4 suggesting that further investigation may be needed, 5 to 6 indicating further investigation and changes required soon, and 7 reflecting an immediate need for investigation and ergonomic intervention.

In the current study, the RULA assessments were performed for three different stages of adrenalectomy,  including, exposure, dissection, and hemostasis and closure. To minimize observer bias, the assessments were performed during the operation by the same research fellows.

### Cost

Cost data were collected from the financial department and categorized into operating room cost, hospital cost, and total cost. These were direct costs per patient allocated out by professional and technical charges. All direct charges had cost relative value units (RVUs) that allocated out expenses posting to that direct center (e.g., salaries-wages-benefits, supplies). The cost comparison included the fixed cost of the robot itself. Per institutional regulations, the costs are reported as a ratio between the groups.

### Statistical Analysis

Descriptive statistical analyses were performed using JMP, version 18.0 software (SAS Institute Inc., Cary, NC, USA). Categorical variables were analyzed using the chi-square test. For continuous variables, the Kolmogorov–Smirnov goodness-of-fit test was performed  to determine the distribution of the data. For equally distributed data, a parametric test (Student’s *t* test) was selected, and for unequally distributed data, a non-parametric test (Mann-Whitney *U* ranked sum test) was used. All tests were two-tailed and performed at a significance level of 0.05. Continuous variables are presented as median (interquartile range [IQR]), and categorical variables are reported as number (%).

## Results

For this study, 27 patients each were randomized to the RA and LA groups. Table 1 summarizes the clinical variables. The groups were similar regarding demographic, clinical, and anthropometric variables. Tumor size was 29.2 mm (IQR, 18–40 mm) versus 32.3 mm (IQR, 18–40.7 mm) (*P* = 0.41), and body mass index (BMI) was 31.7 kg/m^2^ (IQR, 28–36.5 kg/m^2^) versus 31 kg/m^2^ (IQR, 27–39 kg/m^2^) (*P* = 0.33) in the RA and LA groups, respectively. The clinical diagnoses were similar, with mild autonomous cortisol secretion (MACS) as the dominant indication for adrenalectomy in each group. Perirenal  fat thicknesses also were similar (15.3 mm [IQR, 8.9–19.6 mm] in the RA group versus 14.1 mm [IQR, 8.5–24.6 mm] in the LA group; *P* = 0.5).

**Table 1:** Demographic and clinical details of study patients

Parameter	Robotic	Laparoscopic	*P* Value
(*n* = 54)	(*n* = 27) *n* (%)	(*n* = 27) *n* (%)	
Median age: years (IQR)	56 (48–66)	61 (43–70)	0.36
Gender			
Male Female	8 (30) 19 (70)	12 (44) 15 (56)	0.26
Median BMI: kg/m^2^ (IQR)	31.7 (28–36.5)	31 (27–39)	0.33
Tumor side			
Right Left	10 (37) 17 (63)	14 (52) 13 (48)	0.27
Median tumor size: mm (IQR)	29.2 (18–40)	32.3 (18–40.7)	0.41
Diagnosis			
MACS	15 (55)	15 (55)	0.57
Primary aldosteronism	7 (26)	5 (18)	
Pheochromocytoma	3 (11)	3 (11)	
Cushing’s syndrome	1 (4)	2 (8)	
Non-secreting adrenocortical adenoma	0 (0)	1 (4)	
Malignancy^a^	1 (4)	0 (0)	
Myelolipoma^b^	0 (0)	1 (4)	
Median perirenal fat thickness: mm (IQR)^c^	15.3 (8.9–19.6)	14.1 (8.5–24.6)	0.5

IQR, interquartile range; BMI, body mass index; MACS, mild autonomous cortisol secretion

^a^Aggressive B-cell lymphoma metastasis

^b^In the patient with myelolipoma, preoperative imaging studies suggested a possible adrenal malignancy.

^c^Refers to the thickness of retroperitoneal fat around the ipsilateral kidney at the level of the hilum

Table 2 summarizes the perioperative outcomes of the study. The skin-to-skin operative time was similar in the RA and LA groups (101 min [IQR, 89–138 min] vs 112 min [IQR, 89–26 mm]; *P* = 0.47). Door-to-door times, anesthesia times, and individual components of the operation (exposure, dissection, and closure times) also were similar between the groups. The median robotic docking time was 7 min (IQR, 5.5–8.5 min). A similar number of trocars were used in each group, but the RA group required less frequent camera cleaning (2 [IQR, 1–3] vs 5 [IQR, 2–6], respectively; *P* = 0.03) and had less estimated blood loss (5 ml [IQR, 1–10 ml] vs 10 ml [IQR, 5–20 ml], respectively; *P* = 0.03). The capsular disruption rates were the same in the two groups (4% each).

**Table 2:** Perioperative outcomes for study patients

Parameter	Robotic	Laparoscopic	*P* Value
Median operative time: min (IQR)	101 (89–138)	112 (89–126)	0.47
Median door-to-door time: min (IQR)	188 (173–235)	195 (179–227)	0.44
Median anesthesia time: min (IQR)	181 (163–223)	181 (161–200)	0.34
Median positioning time: min (IQR)	10 (8–11)	9 (8–11)	0.32
Median exposure time: min (IQR)	20.5 (13–35)	26 (23–35)	0.09
Median dissection time: min (IQR)	48.5 (42–58)	44 (36–62)	0.21
Median closure time: min (IQR)	26 (19–32.5)	26 (20–40.5)	0.16
Median no. of trocars used (IQR)	5 (5–6)	5 (5–6)	0.26
Camera cleaning count: *n* (%)	2 (1–3)	5 (2–6)	0.03
Hand assistance: *n* (%)	0 (0)	1 (4)	0.24
Conversion to open: *n* (%)	0 (0)	1 (4)	0.24
Readmission: *n* (%)	0 (0)	0 (0)	1
Partial adrenalectomy: *n* (%)	0 (0)	1 (4)	0.24
Case abortion: *n* (%)	0 (0)	1 (4)	0.24
Median estimated blood loss: ml (IQR)	5 (2–10)	10 (5–20)	0.03
Capsular disruption: *n* (%)	1 (4)	1 (4)	1
Median hospital stay: days (IQR)	1 (1–1)	1 (1–1)	0.42
Median POD1 pain score (IQR)	7 (6–8)	7 (6–8)	0.49
Median POD2 pain score (IQR)	6 (4–7)	5 (4–7)	0.43
Median POD14 pain score (IQR)	2 (1–3)	2.5 (1–4)	0.20
Median total morphine-equivalent dose: mg (IQR)	15 (6.7–20)	8.3 (5–16.5)	0.11
Postoperative day 30 complications: *n* (%)	1 (4)	0 (0)	0.24
Median primary surgeon NASA-TLX score (IQR)	16 (10–36)	48 (36–66)	<0.001
Median assistant surgeon NASA-TLX score (IQR)	49.3 (42.7–53.3)	52 (47–64)	0.16
Median primary surgeon RULA score (during exposure) (IQR)			
Left Right	6 (5–6) 5 (5–6)	6 (5–6) 6 (5–6)	0.16 0.04
Median assistant surgeon RULA score (during exposure) (IQR)			
Left Right	5 (5–6) 6 (5–6)	6 (5–6) 6 (5–6)	0.20 0.18
Median primary surgeon RULA score (during dissection) (IQR)			
Left Right	3 (3–3) 3 (3–3)	6 (5–6) 6 (5–6)	<0.001 <0.001
Median assistant surgeon RULA score (during dissection) (IQR)			
Left Right	5 (4–6) 5 (4–5)	6 (6–6) 6 (6–6)	<0.001 <0.001
Primary surgeon RULA score (during hemostasis & closure), median (IQR)			
Left Right	5 (5–6) 5 (5–6)	5 (5–6) 6 (5–6)	0.30 0.16
Median assistant surgeon RULA score (during hemostasis & closure) (IQR)			
Left Right	6 (5–6) 5 (4–6)	5 (5–6) 5 (5–6)	0.21 0.46
Median primary surgeon RULA (total) (IQR)			
Left Right	14 (13–15) 14 (13–14)	17 (16–18) 17 (17–18)	<0.001 <0.001
Median assistant surgeon RULA (total) (IQR)			
Left Right	16 (14–18) 16 (14–17)	17 (16–18) 17 (16–18)	0.04 0.03

IQR, interquartile range; POD, postoperative day; NASA, National Aeronautics and Space Administration; TLX, Task Load Index; RULA, rapid upper limb assessment

All the cases were completed as planned in the RA group, but the LA group had one conversion to open surgery (4%), one conversion to hand-assisted surgery (4%), one abortion of the case (4%), and one conversion to partial adrenalectomy because of inability to continue the dissection safely and efficiently (4%), adding up to a 15% (*n* = 4) rate of deviation from the original minimally invasive adrenalectomy plan in the LA group compared with 0% deviation in the RA group (*P* = 0.02). The aborted case had a significant inflammation in the retroperitoneum, creating indistinct tissue planes during attempted adrenalectomy for MACS caused by a radiologically benign adrenal nodule. Because the laparoscopic two-dimensional view did not display the necessary tissue distinction and rigid instruments did not provide the required angle of dissection for an ability to proceed safely with adrenalectomy, it was decided to abort the surgical procedure and treat the patient medically instead. Because the robot was not available, a robotic conversion could not be performed.

The postoperative pain scores and morphine-equivalent doses were similar between the groups (Table 2). No mortality occurred in either group, whereas, morbidity was detected in one patient as a persistent abdominal pain requiring an emergency room visit (4%) in the robotic group and in none of the laparoscopic patients.

The operating surgeon NASA-TLX score and overall RULA score were lower in the robotic versus laparoscopic group (overall NASA-TLX score: 16 [IQR, 10–36] vs 48 [IQR, 36–66], *P ≤* 0.001; RULA left side: 14 [IQR, 13–15] vs 17 [IQR, 16–18], *P* = 0.001; RULA right side: 14 [IQR, 13–14] vs 17 [IQR, 17–18], respectively, *P ≤* 0.001). The assistant surgeon’s NASA TLX score was similar in both groups, but the RULA score was lower in the RA group than in the LA group (Table 2).

Table 3 provides a breakdown of the NASA TLX scores across different categories. For the operating surgeon, the main challenge was mental in the robotic procedures, but physical and effort-related in the laparoscopic procedures. The assistant surgeon felt a similar amount of challenge regarding the different components in creating the workload of robotic versus laparoscopic adrenalectomy, except for a greater effort challenge in the laparoscopic procedures. The operating room, hospital, and total costs were similar between the RA and LA groups (Table 4).

**Table 3:** Detailed NASA-TLX scores in each group

NASA-TLX subsections	Median robotic (IQR)	Median laparoscopic (IQR)	*P* Value
Primary surgeon Mental Demand	80 (40–120)	30 (0–50)	<0.001
Primary surgeon Physical Demand	30 (0–100)	240 (50–360)	<0.001
Primary surgeon Temporal Demand	35 (20–75)	55 (15–70)	0.40
Primary surgeon Performance Demand	0 (0–0)	75 (60–125)	<0.001
Primary surgeon Effort Demand	75 (30–105)	220 (120–280)	<0.001
Primary surgeon Frustration Demand	5 (0–15)	100 (0–180)	<0.001
Primary surgeon weighted score	16 (10–36)	48 (36–66)	<0.001
Assistant surgeon Mental Demand	70 (20–180)	80 (40–180)	0.29
Assistant surgeon Physical Demand	165 (80–240)	180 (70–280)	0.31
Assistant surgeon Temporal Demand	80 (40–150)	45 (20–150)	0.14
Assistant surgeon Performance Demand	100 (75–150)	110 (75–150)	0.44
Assistant surgeon Effort Demand	210 (160–260)	240 (195–320)	0.03
Assistant surgeon Frustration Demand	0 (0–15)	0 (0–0)	0.26
Assistant surgeon weighted score	49.3 (42.7–53.3)	52 (47–64)	0.16

NASA, National Aeronautics and Space Administration; TLX, Task Load Index; IQR, interquartile range

**Table 4:** Cost analysis of the groups

Cost	Robotic-to-laparoscopic ratio	*P* Value
Total cost	1.01	0.52
Operating room cost	0.97	0.17
Hospital cost	1.17	0.42

## Discussion

To the best of our knowledge, this study is the most comprehensive study comparing LA and RA performed using the most recent technologies in a randomized design. The results suggest that in experienced hands, the primary outcome, operative time, and other secondary perioperative outcomes, including costs, are similar between LA and RA, with less physical burden on the surgical team with the robotic approach. Furthermore, the rate of deviations from the original minimally invasive adrenalectomy plan was higher in the laparoscopic group (15%) than in the robotic group (0%) in terms of conversions to open, hand-assisted approach, partial adrenalectomy or abortions. These deviations occurred due to the inability to continue the dissection safely and efficiently in the laparoscopic group related to challenging patient and tumor anatomy.

Laparoscopic adrenalectomy is an excellent choice for removing adrenal tumors, and as the study showed, works perfectly in most cases. However, in the presence of difficult patient anatomy and dissection planes, with laparoscopic rigid instruments approaching the tumor in a parallel, rather than an angled fashion, the chances of completing the procedure in a minimally invasive manner decrease with the laparoscopic approach, especially if there is also abundant retroperitoneal fat and inflammation. However, the articulating wristed instrumentation with robotic systems decreases the impact of these adverse factors on the performance of the procedure. Although exceptions exist, it seems that the robotic rather than the laparoscopic approach would be more appropriate for patients with adverse preoperative predictors such as abundant retroperitoneal fat, periadrenal inflammation, large (≥ 5 cm) tumors, large liver, large spleen, and blood vessel proximity.

The description of laparoscopic transabdominal lateral adrenalectomy by Gagner et al.^17^ in 1992 and posterior retroperitoneal adrenalectomy by Mercan et al.^18^ in 1993 revolutionized the surgical management of adrenal tumors by allowing the patients to enjoy the benefits of minimally invasive surgery instead of enduring the morbid recovery of open surgery. Subsequent studies performed on large national quality databases documented the impact by showing a shorter operative time, a shorter hospital stay, and a lower morbidity rate with laparoscopic versus open surgery.^19,20^

Until recently, the laparoscopic approach has been recognized as the “gold standard” for removing most adrenal tumors, with controversies centered on the choice of a lateral transabdominal versus a posterior retroperitoneal approach.^21^ Nevertheless, robotic systems were introduced into general surgical procedures at the end of 1990s and received significant attention due to associated technologic novelties, incorporation of articulating wristed instrumentation, a 3D view, and a computer-assisted remote interface.^22,23^

The first randomized study comparing robotic and laparoscopic adrenalectomies was reported by Morino et al.^6^ in 2004. In this study of 20 randomized patients, the authors found that laparoscopic versus robotic adrenalectomy was associated with a shorter operative time, lower cost, and fewer perioperative comorbidities. In addition, 4 of the 10 patients in the robotic group were converted to laparoscopic surgery. Subsequently, Ma et al.^7^ reported the second randomized study in 2020, which included only pheochromocytoma patients. In this study, robotic versus laparoscopic adrenalectomy was faster and associated with less blood loss, but was more expensive. The findings of the current study differ from the results of these two studies. The differences probably are related to differences in experience, quality of robotic systems, and tumor types treated.

The current study had several weaknesses. First, it involved one attending surgeon (E.B.). Although, this design excluded the bias of surgeon heterogeneity and its effect on outcomes, it created challenges in generalizing results to lower-volume surgeons. Second, the percentage of more challenging adrenalectomies (patients with a BMI ≥ 35 kg/m^2^, a tumor size ≥ 5 cm, pheochromocytomas, and malignancies) was low in the overall study population, which may have prevented the separation of outcomes. We aim to provide more more data regarding these more challenging adrenalectomies in future phases of this study. 

The higher cost of robotic versus laparoscopic surgery is frequently criticized.^24,25^ This study is one of the few studies to date showing a similar cost between the two approaches.^26^ This was achieved by the omission of disposables (e.g., clip appliers and staplers) due to the ease of intracorporeal knot-tying, even with difficult angles, better optics, and hence less need for intraoperative ultrasound and fewer conversions from the original minimally invasive plan with the robotic approach.

The fourth arm of the robot is critically important to the utilization of all the features of this advanced surgical system. We have used it to provide a very effective counter-traction of the adrenal gland to facilitate the dissection, which ultimately  increased the safety and efficacy of the procedure. As a result, for right-side lesions, we have preferred to use a stationary laparoscopic liver retractor rather than the fourth arm to retract the liver.

Indocyanine green (ICG) has been shown to provide a tissue contrast distinction between the fluorescent adrenal tissue and the non-fluorescent retroperitoneum.^27^ Due to availability of this technology in the robotic system, ICG imaging was used routinely in the robotic arm of the study. However, because the dye was given by the anesthesia team concurrently during the procedure and the ICG view was used for a few seconds intraoperatively, it did not prolong the robotic cases. It was not used in the laparoscopic cases because the laparoscopic towers used did not have this capability.

In summary, this randomized study on robotic versus laparoscopic adrenalectomy using a modern technology showed similar length of operative time, perioperative outcomes, and cost, with a lower physical burden on the operating surgeon and less chance of deviation from the original minimally invasive surgical plan in the robotic versus laparoscopic group.

## Supplementary Information

Below is the link to the electronic supplementary material.

Supplementary file1 (DOC 71 kb)
