# Correction: Laparoscopic Versus Robotic Adrenalectomy: A Randomized Clinical Trial

**DOI:** 10.1245/s10434-025-18780-x

**Published:** 2025-12-01

**Authors:** Eren Berber, Arturan Ibrahimli, Edip Memisoglu, Ege Akgun, Rafael Perez-Soto

**Affiliations:** Center for Endocrine Surgery, Cleveland Clinic, Cleveland, OH USA

**Correction: Annals of Surgical Oncology** 10.1245/s10434-025-18567-0

In the original online version of this article, there were errors in Table 1. The values for pheochromocytoma and primary aldosteronism diagnoses were incorrect.

The original article was corrected.

