# Supplementary material for: Laparoscopic Versus Robotic Adrenalectomy: A Randomized Clinical Trial
Source: Ann Surg Oncol. 2025 Oct 25;33(2):1319–28. doi: 10.1245/s10434-025-18567-0 (PMC12765717; doi:10.1245/s10434-025-18567-0)
Supplement: Supplementary file 1 — Supplementary file1 (DOC 71 kb) [file 10434_2025_18567_MOESM1_ESM.doc]

**Study Title:** LAPAROSCOPIC VERSUS ROBOTIC LATERAL TRANSABDOMINAL ADRENALECTOMY: A SINGLE-CENTER RANDOMIZED PROSPECTIVE TRIAL

**Principal Investigator**: Eren Berber,MD

**Sponsor, if Applicable (include NIH and other funding sources here)**:None

1. **Background and Significance**

Adrenalectomy used to be done through big open chevron, subcostal or thoraco-abdominal incisions that led to significant recovery and morbidity. In 1990s, the description of laparoscopic adrenalectomy revolutionized the care of these patients by converting the procedure into a minimally invasive operation with a short hospital stay and recovery. Since then, many centers have reported the safety and efficacy of laparoscopic adrenalectomy. Laparoscopic surgery uses rigid, straight instruments operated by the surgeons under the visual guidance of a two-dimensional video platform.

In the late 2000s, robotic systems have been developed that incorporated articulating wristed instruments used with a three-dimensional computerized video platform. Over the past two decades, robotic systems have penetrated many thoracic, cardiac and abdominal procedures.

A review of the National inpatient database in 2016 showed that 32.7% of the adrenalectomies in the US are being done robotically and 48.5% laparoscopically. Nevertheless, there are scant comparative data and only two randomized studies comparing laparoscopic with robotic adrenalectomy, one of which suffers from a small sample size (10 patients in each group Morino et al Surg Endosc) and the other from exclusion of tumor types (pheochromocytoma only Ma W et al Eur J Surg Oncol). The first study found laparoscopic approach to be superior and the latter study robotic approach to be more advantageous. Both studies highlighted the cost of robotic surgery to be a disadvantage versus laparoscopic approach. Underscoring the lack of data to recommend one technique versus the other, a meta-analysis concluded that robotic adrenalectomy is a safe and feasible procedure with similar clinical outcomes as the laparoscopic approach and recommended high quality randomized clinical trials to determine whether laparoscopic vs robotic approach was superior to perform adrenalectomy.

Our group has a high-volume minimally invasive adrenalectomy program with a good mixture of laparoscopic and robotic surgical expertise, performing close to 100 surgical cases a year. There are a number of barriers to performing the randomized studies required for adrenalectomy. The first one is the adrenal surgery volume. An average general surgeon does one adrenal surgery a year. A high-volume adrenal surgeon is considered to do > 4-6 adrenalectomies a year. Furthermore, there are only a few centers in the world that possesses a large both laparoscopic and robotic adrenalectomy experience. Being a unique adrenal surgery center, we believe that our center is one of the few centers in the world qualified to perform a randomized clinical trial comparing laparoscopic with robotic adrenalectomy. We believe that such a study will help understand whether one approach is more advantageous over the other regarding surgical outcomes, especially with the increasing use of robotics in surgical procedures.

1. **Specific Aims**

The aim is to investigate whether there are any differences between laparoscopic and lateral transabdominal adrenalectomy in terms of perioperative outcomes and surgeon ergonomics. The study is expected to generate data that will help answer the question of whether laparoscopic and robotic approaches to adrenalectomy have any advantages to each other. This will help improve patient care, patient outcomes and maximize the appropriate utilization of resources in adrenal surgery.

1. **Study Design**

Study Design

Prospective randomized study

Subject Population

Patient inclusion criteria:

1. Men and women between ages 18 and 75
2. Diagnosis of an adrenal tumor/pathology planned for a minimally invasive adrenalectomy at the department of endocrine surgery at the Cleveland Clinic.

Patient exclusion criteria:

1. Requirement for an open adrenalectomy based on imaging studies suggesting an aggressive cancer.
2. The presence of extensive surgical history precluding a minimally invasive approach to be undertaken.
3. Patients planned for a partial, rather than a complete adrenalectomy, as the former is a much easier procedure.
4. Patients planned for a posterior adrenalectomy (these would be patients with an extensive surgical history with significant intra-abdominal adhesions and those requiring bilateral adrenalectomy).
5. Mental incapacity or language barrier
6. Any condition, unwillingness, or inability, not covered by any of the other exclusion criteria, which, in the study clinician’s opinion, might jeopardize the subject’s safety or compliance with the protocol

As surveys will be done on the surgical team, the surgeons are also subjects of this study. An information letter will be provided to them and those wishing their data not collected will be excluded from the study. They will be given Surgeon mental load using NASA Task Load Index Scale and

Surgeon ergonomics as assessed by Rapid Upper Limb Assessment (RULA) surveys

Study Recruitment

The recruitment will be done at the office visits when the patients are consented for the surgical procedure. An informed consent form will be presented to the patients by the research team and the patients will be given the opportunity to participate in this study.

Study Procedures

# The patients planned for a minimally invasive lateral transabdominal adrenalectomy will be approached by the PI at the time of their office visit for an opportunity to participate in this research study. If the patients agree to participate, the consent form will be presented to them and any questions will be answered. Other than the data collection, the management of the patients throughout their whole care will be as part of standard of care. They will undergo the minimally invasive adrenalectomy (laparoscopic or robotic based on randomization) and when they are ready to be discharged, they will be discharged home. The procedures will be done as part of standard of care using well-established laparoscopic and robotic techniques. These two techniques are identical procedures, with the same patient positioning, similar number and size of incisions, flow of the procedure and postoperative care, expect that in the laparoscopic procedure, the procedure will be done using standard laparoscopic equipment and in the robotic procedure, with the robotic platform.

# Then, as part of routine care, they will be seen in the clinic for postoperative check at 2 weeks. Those with pheochromocytomas or malignancy, will follow the standard of care follow up at 6 months. Other than the randomization into a laparoscopic or robotic procedure, there are no extra labs, imaging studies or protocols.

The primary outcome is-skin-to skin operative time. Secondary outcomes are:

1. Conversion to open, robotic or laparoscopic approach
2. Estimated blood loss
3. Postoperative pain scores (according to visual assessment score 0-10, with o being non pain and 10 being worst pain)
4. Hospital stay
5. Cost, which will be reported as reported in previous similar CCF randomized studies (Petro et al. robotic versus laparoscopic ventral hernia repair, JAMA Surgery 2021) as disposable/reusable median cost ratio and operating room time-cost ratio.
6. Postoperative morphine equivalent opioid consumption
7. Perioperative 30-day complications
8. Surgical margin clearance for malignant tumors and pheochromocytoma
9. Surgeon mental load using NASA Task Load Index Scale
10. Surgeon ergonomics as assessed by Rapid Upper Limb Assessment (RULA) survey

# Recruitment Procedures **(*All recruitment material must submitted for IRB approval)***

The recruitment will be done at the office visits when the patients are consented for the surgical procedure. An informed consent form will be presented to the patients by the research team (PI) and the patients will be given the opportunity to participate in this study. Any questions of the patients related to the study will be answered before signing the consent. Our practice involves adult patients.

Once the surgical teams are assigned, the members of the surgical team will be approached by the PI (Eren Berber) regarding participation and given the information sheet.

If the surgeons express that they do not want their data to be collected, they will not be administered the surveys.

# **Data Analysis Plan**

# Data Collection:

The data will be recorded in real time in the operating room by the Endocrine Surgery Research fellows who will be trained for data collection specific to this study. The data that was recorded on the forms will be entered into a RedCap registry. The original source data forms will also be kept in a binder at the PI’s locked office and then transitioned to the EMI clinical research offices. The PI will be responsible for the privacy and safety of data collection. The data forms will be stored for **2 years** after study closure.

Power Analysis:

A review of the adrenalectomy database at the Endocrine Surgery department revealed a mean operative time of 147.4 minutes and a standard deviation of 37.9. A sample size calculation using an alpha of 0.05, beta of 0.2, 50%/50% proportions, hypothesizing a 30-minute difference in operative time with a robotic vs laparoscopic approach using the historical Standard deviation of 37.9 revealed 27 patients in each group, for a total of 54 patients. Since the study does not involve follow up participation, other than the immediate follow up, a subject drop off component was not added to the sample size.

# Statistical Analysis

# The data will be recorded in real time in the operating room by the Endocrine Surgery Research fellows who will be trained for data collection specific to this study. The data forms will be entered into a RedCap registry. Statistical analysis will be performed using Mann Whitney U test and Chi square tests, as deemed appropriate using JMP software. A p value of < 0.05 will be accepted as statistically significant. The statistical analyses that will be performed by the PI, who has taken the Cleveland Clinic statistics course and Case Western Statistics class as part of the masters in business administration program, will be reviewed by the Cleveland Clinic Statistics Department (James Bena being the Department Liason).

# Randomization Method

# Parallel groups of patients will be randomly allocated in a 1:1 randomization to laparoscopic or robotic surgery.

# **Alternatives**

# The study subjects will be presented with the two standard of care options, laparoscopic or robotic transabdominal lateral adrenalectomy. These two options are the current options for the treatment of these patients. The design of the study allows for a conversion of the procedure to either other technique (I,e. from laparoscopic to robotic, or robotic to laparoscopic) based on the conduct of the operation and therefore, there will be no deviation from standard or care or any restriction of the access of the patient to available technologies if there is a clinical need at the time of the randomized procedure.

# The only alternative is not to participate. In that case, the adrenalectomy procedure will be performed laparoscopically or robotically based on availability of equipment and discretion of the surgeon.

# **Risks**

# There are no additional risks imposed to the patients on top of expected perioperative surgical risks from a minimally invasive adrenalectomy as a result of their participation in the study. There is a potential risk of loss of confidentiality. This will be minimized by entering into and storing data at the secure website Redcap, and limiting access to the data to study team (PI, study research fellows and coordinators).

# **Benefits**

There is no benefit associated with participation to the patients. However, the study is expected to generate data that will help answer the question of whether laparoscopic and robotic approaches to adrenalectomy have any advantages to each other. This will help improve patient care, patient outcomes and maximize the appropriate utilization of resources in adrenal surgery.

# **Costs**

# There are no additional costs to the patients as part of their participation in the study. Other than the patients being randomized to a laparoscopic or robotic procedure, all of the study interventions/tests/clinical evaluations/follow up are part of standard of care.

# **Compensation**

# There is no compensation for participation in this trial.

# **Privacy and Confidentiality**

# The data will be maintained in Redcap. The study forms filled out in the operating room and the consent forms will be kept in the PIs locked office at the Department of Endocrine Surgery without any sharing with any other investigators. The PI, research fellows and coordinators will have access to the data and Redcap preferences will be set up to prevent any PHI from Redcap to be extracted. The patients will be assigned a unique study number.

# **DATA/SAMPLE SHARING**

# The data will not be released to any party or entity outside the design of this study. The data forms collected in the operating room will be entered the same day to Redcap by the participating research fellows to ensure accuracy.

# **Adverse Events and Data Monitoring Committee (DMC)**

As there are no interventions that are not part of standard of care in the study, and the team is very experienced with both laparoscopic and robotic adrenalectomy, a DMC will not be used. However, if any adverse event, not observed within our standard practice, is encountered, this will be captured by the research team, recorded in data forms and reported to IRB in writing. An interim analysis will be performed when 50% of the study has been concluded.

1. **ADDITIONAL DOCUMENTS**

See consent form attached.

**ETHICAL BACKGROUND**

Our first and foremost principle in the design of the study is to make sure the patients are not placed in a situation restricting their access to the most appropriate surgical technology for their tumors as a result of randomization. According to the literature and also in our experience, both laparoscopic and robotic approaches are acceptable options for delivering a standard of care to the care of these patients. Our team is also very experienced in performing adrenalectomy using both types of surgical equipment. Still, to make sure the patients are not deprived of any advanced technology, for those patients randomized to the laparoscopic arm, the robotic equipment will be made available in the room and there will be an option to switch to the robotic procedure if any shortcomings of the laparoscopic technique, such as inability provide the adequate safe exposure for a certain surgical task due to body habitus, i.e. excessive obesity, or tumor characteristics for the performance of adrenalectomy, and inability to perform the dissection with a reasonable rate of progression. These requirements for a conversion to the robotic technique will be recorded as a study parameter. Likewise, in the robotic arm, there will be an option to switch to a laparoscopic approach if the same limitations for the robotic approach are experienced by the operating team. This design eliminates any comprise in the use of any necessary technology in a given patient due to the randomization.

1. **REFERENCES**
2. M. Morino, G. Beninca` , G. Giraudo, G. M. Del Genio, F. Rebecchi, C. Garrone. Robot-assisted vs laparoscopic adrenalectomy: A prospective randomized controlled trial. Surg Endosc (2004) 18: 1742–1746.
3. Ma W, Mao Y, Zhuo R, Dai J, Fang C, Wang C, Zhao J, He W, Zhu Y, Xu D, Sun F. Surgical outcomes of a randomized controlled trial compared robotic versus laparoscopic adrenalectomy for pheochromocytoma. Eur J Surg Oncol. 2020 Oct;46(10 Pt A):1843-1847. doi: 10.1016/j.ejso.2020.04.001. Epub 2020 May 23.

# Sung TY, Tennakoon L, Alobuia WM, Seib C, Cisco R, Lin D, Kebebew E. Factors associated with postoperative complications and costs for adrenalectomy in benign adrenal disorders. Surgery. 2022 Jun;171(6):1519-1525. doi: 10.1016/j.surg.2021.10.065. Epub 2021 Nov 29.

# Piccoli M, Pecchini F, Serra F, Nigro C, Colli G, Gozzo D, Zirilli L, Madeo B, Rochira V, Mullineris B. Robotic Versus Laparoscopic Adrenalectomy: Pluriannual Experience in a High-Volume Center Evaluating Indications and Results. J Laparoendosc Adv Surg Tech A. 2021 Apr;31(4):375-381. doi: 10.1089/lap.2020.0839. Epub 2021 Jan 15.

1. Economopoulos KP, Mylonas KS, Stamou AA, Theocharidis V, Sergentanis TN, Psaltopoulou T, Richards ML.  [Laparoscopic versus robotic adrenalectomy: A comprehensive meta-analysis.](https://pubmed.ncbi.nlm.nih.gov/28043926/) Int J Surg. 2017 Feb;38:95-104. doi: 10.1016/j.ijsu.2016.12.118. Epub 2016 Dec 30.
2. Yip L, Duh QY, Wachtel H, Jimenez C, Sturgeon C, Lee C, Velázquez-Fernández D, Berber E, Hammer GD, Bancos I, Lee JA, Marko J, Morris-Wiseman LF, Hughes MS, Livhits MJ, Han MA, Smith PW, Wilhelm S, Asa SL, Fahey TJ 3rd, McKenzie TJ, Strong VE, Perrier ND. American Association of Endocrine Surgeons Guidelines for Adrenalectomy: Executive Summary.JAMA Surg. 2022 Oct 1;157(10):870-877. doi: 10.1001/jamasurg.2022.3544.
3. Hue JJ, Ahorukomeye P, Bingmer K, Drapalik L, Ammori JB, Wilhelm SM, Rothermel LD, Towe CW. A comparison of robotic and laparoscopic minimally invasive adrenalectomy for adrenal malignancies. Surg Endosc. 2022 Jul;36(7):5374-5381. doi: 10.1007/s00464-021-08827-x. Epub 2021 Nov 1.PMID: 34724582
4. Petro CC, Zolin S, Krpata D, Alkhatib H, Tu C, Rosen MJ, Prabhu AS. Patient-Reported Outcomes of Robotic vs Laparoscopic Ventral Hernia Repair With Intraperitoneal Mesh: The PROVE-IT Randomized Clinical Trial. JAMA Surg. 2021 Jan 1;156(1):22-29. doi: 10.1001/jamasurg.2020.4569.
5. Ajita S. Prabhu, MD; Alfredo Carbonell, DO; William Hope, MD; Jeremy Warren, MD; Rana Higgins, MD; Brian Jacob, MD; Jeffrey Blatnik, MD; Ivy Haskins, MD; Hemasat Alkhatib, MD; Luciano Tastaldi, MD; Aldo Fafaj, MD; Chao Tu, MS; Michael J. Rosen, MD. Robotic Inguinal vs Transabdominal Laparoscopic Inguinal Hernia Repair The RIVAL Randomized Clinical Trial. AMA Surg. 2020 May 1;155(5):380-387. doi: 10.1001/jamasurg.2020.0034.
